# Analysis of the ORFK1 hypervariable regions reveal distinct HHV-8 clustering in Kaposi’s sarcoma and non-Kaposi’s cases

**DOI:** 10.1186/s13046-014-0119-0

**Published:** 2015-01-16

**Authors:** Paola Cordiali-Fei, Elisabetta Trento, Marta Giovanetti, Alessandra Lo Presti, Alessandra Latini, Massimo Giuliani, Giovanna D’Agosto, Valentina Bordignon, Eleonora Cella, Francesca Farchi, Carmela Ferraro, Ilaria Lesnoni La Parola, Carlo Cota, Isabella Sperduti, Antonella Vento, Antonio Cristaudo, Massimo Ciccozzi, Fabrizio Ensoli

**Affiliations:** Clinical Pathology and Microbiology, San Gallicano Dermatology Institute, Via Elio Chianesi 53, 00144 Rome, Italy; Department of Infectious Parasitic and Immunomediated Diseases, National Institute of Health, Viale Regina Elena 299, 00161 Rome, Italy; Infective Dermatology, San Gallicano Dermatology Institute, Via Elio Chianesi 53, 00144 Rome, Italy; Clinical Dermatology, San Gallicano Dermatology Institute, Via Elio Chianesi 53, 00144 Rome, Italy; Dermopathology, San Gallicano Dermatology Institute, Via Elio Chianesi 53, 00144 Rome, Italy; Epidemiology, Regina Elena Cancer Institute, Via Elio Chianesi 53, 00144 Rome, Italy; University of Biomedical Campus, Via Álvaro del Portillo, 200, 00128 Rome, Italy

**Keywords:** HHV-8, Kaposi’s sarcoma, Phylogenesis, HHV-8 variants, Selective pressure

## Abstract

**Background:**

Classical Kaposi’s Sarcoma (cKS) is a rare vascular tumor, which develops in subjects infected with Human Herpesvirus-8 (HHV-8). Beside the host predisposing factors, viral genetic variants might possibly be related to disease development. The aim of this study was to identify HHV-8 variants in patients with cKS or in HHV-8 infected subjects either asymptomatic or with cKS-unrelated cutaneous lymphoproliferative disorders.

**Methods:**

The VR1 and VR2 regions of the ORF K1 sequence were analyzed in samples (peripheral blood and/or lesional tissue) collected between 2000 and 2010 from 27 subjects with HHV-8 infection, established by the presence of anti-HHV-8 antibodies. On the basis of viral genotyping, a phylogenetic analysis and a time-scaled evaluation were performed.

**Results:**

Two main clades of HHV-8, corresponding to A and C subtypes, were identified. Moreover, for each subtype, two main clusters were found distinctively associated to cKS or non-cKS subjects. Selective pressure analysis showed twelve sites of the K1 coding gene (VR1 and VR2 regions) under positive selective pressure and one site under negative pressure.

**Conclusion:**

Thus, present data suggest that HHV-8 genetic variants may influence the susceptibility to cKS in individuals with HHV-8 infection.

## Background

Human Herpesvirus-8 (HHV-8) infection is associated with three human malignancies: Kaposi’s sarcoma (KS), primary effusion lymphoma (PEL), and multicentric Castleman’s disease (MCD) [1]. KS is a vascular tumor thought to arise from HHV-8 infected cells of endothelial origin, whereas PEL and MCD appear of B-cell origin [2]. However, HHV-8 may also cause a persistent infection without clinical symptoms [3] or characterized by an acute inflammatory syndrome [4,5]. A significant high rate of HHV-8 infected subjects was also found among patients with cutaneous lymphoproliferative diseases [6,7], characterized by the proliferation of T lymphocytes showing different grades of tropism for the epidermal layer of the skin, as the early stage Mycosis Fungoides (eMF) [8] or the cutaneous T cell lymphoma Mycosis Fungoides (MF) [9].

Although HHV-8 infection alone might not represent the primary cause for KS development [10], the pathogenic processes leading to KS development correlate with HHV-8 reactivation from latency, increased viral load and production of viral factors, which interfere with host cellular functions and allow the virus to escape from the host immune response [11]. Additionally, host predisposing factors appear to play a part in promoting the disease, including either immune suppression or an unbalanced immune response [11-13]. In fact, either an association with HIV [14,15] or EBV co-infections [15-17] and a genetic background involving genes coding for inflammatory cytokines [18,19] or cell cycle control proteins [20] represent high risk factors for KS development. On the other hand, HHV-8 itself might have oncogenic potentials, since multiple viral genes can regulate pathways controlling the switch between latent and lytic replication [21] and alter intracellular signaling pathway important in regulating the cell cycle as PI3K/AKT/MTOR [22]. In fact, the HHV-8 genome contains 87 open reading frames (ORFs) that encode homologues to cellular proteins involved in signal transduction, cell cycle regulation, and/or inhibition of apoptosis. All of them play key roles in cell growth and might be potentially involved in cell transformation [2]. Emerging evidence suggests that the HHV-8 ORF74 gene, which encodes the viral G protein-coupled receptor (vGPCR), may play a part in KS development [23,24]. However, other genes might possibly be implicated in cell transformation [2,25,26], including ORF-K1, encoding a transmembrane glycoprotein (K1) expressed by infected cells, with a cytoplasmic tail containing a constitutively active immunoreceptor tyrosine activation motif (ITAM), which transduces extracellular signals to elicit cellular activation [27,28]. Moreover, K1 appears to be involved in endothelial cell immortalization [29] and its expression in epithelial and endothelial cells results in the production of vascular endothelial growth factor (VEGF) and matrix metalloproteinase MMP-9 [30], which can favor tumor angiogenesis and tumor cell growth in vivo [31,32].

Sequence analysis of the highly variable ORF K1 regions has allowed the identification of four main HHV-8 subtypes (A, B, C, D) [33] presenting a different distribution in the world: subtype A and C predominate in Europe and USA, while B subtype predominates in Africa and D is present in the Pacific islands [34]. In addition, recently identified subtypes include E, found to be predominant in ancient populations, like Brazilian Amerindians [35], Z, detected in Zambian children [36] and F, identified in Ugandan Bantu tribe [37]. Although viral genetic variants have been suggested to be related to disease activity [38,39], it is still unclear whether different genotypes are associated with diverse clinical settings or rates of KS progression. The aim of this study was to investigate HHV-8 variants, through the analysis of ORF K1 sequences, including the variable regions VR1 and VR2, in patients with cKS or in HHV-8 infected subjects either without clinical symptoms or presenting cutaneous proliferative disorders, including MF or eMF. We further analyzed the nonsynonymous nucleotide mutations in the K1 protein-coding sequence to identify possible molecular variants associated with KS development.

## Methods

### Design of the study

A retrospective study to assess HHV-8 genetic variants in cKS patients and HHV-8-infected subjects either asymptomatic or with cutaneous proliferative disorders (MF or EsMF) was planned. Lesional tissue specimens or frozen peripheral blood mononuclear cells (PBMCs), collected, upon informed consent, between 2000 and 2010 were employed for ORFK1 gene amplification, nucleotide sequencing, phylogenetic analysis and analysis of nonsynonymous mutations. The study was approved by the Institutional Ethical Committee ( Prot. IFO/CE 1352/02).

### Subjects and samples

Table 1 summarizes the clinical and demographic characteristics of the study subjects. The study was performed on a total of 27 subjects, 21 male and 6 female, aged between 38 and 87 yrs (median age: 68 yrs). All originated from central (Abruzzo, Lazio) or south (Calabria, Campania) Italy. Four subjects were clinically asymptomatic, 15 were affected by cKS, 8 presented a cutaneous lymphoproliferative diseases (4 had diagnosis of eMF and 4 of MF, according the WHO-EORTC classification) [8,9]. Staging of KS was performed according to the localization and vascularisation of skin lesions [40], as well as the occurrence of visceral involvement [38]. Clinical diagnosis were confirmed by histological analysis in all patients. Tissue and PBMC samples employed to analyze the HHV-8 ORF K1 sequence were collected between 2000 and 2010, and kept stored at −80°C.

**Table 1:** **Characteristics of the patients and reference subjects (n = 27)**

**Sample ID**	**Material**	**Diagnosis**	**Origin**	**Year of sampling**	**Age**	**Sex**	**Genotype**	**Plasma viral load gen eq/mL**	**Clinical stage**
26 K	Biopsy	cKS	Central Italy	2008	73	M	A	107	B IV
30 K	Biopsy	cKS	South Italy	2008	78	M	A	33	B IV
33 K	Biopsy	cKS	South Italy	2008	65	M	A	563	B IV
36 K	Biopsy	cKS	South Italy	2009	61	F	A	100	B IV
40 K	Biopsy	cKS	Central Italy	2010	38	F	A	Undetectable	A II
47 K	Biopsy	cKS	South Italy	2003	68	M	A	105	B IV
48 K	Biopsy	cKS	South Italy	2003	77	M	A	190	B IV
4 K	Biopsy	cKS	South Italy	2003	72	F	A	100	B II
15 K	Biopsy	cKS	South Italy	2004	68	M	A	57	B IV
18 K	Biopsy	cKS	Central Italy	2005	66	M	C	70	B IV
20 K	Biopsy	cKS	Central Italy	2007	66	M	C	100	A II
21 K	Biopsy	cKS	Central Italy	2007	67	M	C	231	B I
27 K	Biopsy	cKS	Central Italy	2008	87	M	C	5	B IV
34 K	Biopsy	cKS	South Italy	2009	56	M	C	20	A I
37 K	Biopsy	cKS	Central Italy	2009	82	M	C	400	B III
14 K	PBMC	Early MF	Central Italy	2010	41	M	A	Undetectable	
3 K	Biopsy	Early MF	South Italy	2002	56	M	A	Undetectable	
77 K	Biopsy	Early MF	Central Italy	2007	74	M	A	Undetectable	
78 K	Biopsy	Early MF	Central Italy	2006	66	M	A	Undetectable	
17 K	PBMC	MF	Central Italy	2005	70	M	A	124	
13 K	Biopsy	MF	South Italy	2010	72	M	C	21200	
42 K	Biopsy	MF	Central Italy	2010	66	M	C	100	
5 K	Biopsy	MF	Central Italy	2003	40	M	C	125	
1 K	PBMC	Asymptomatic	South Italy	2000	79	F	A	Undetectable	
2 K	PBMC	Asymptomatic	South Italy	2000	83	F	A	Undetectable	
43 K	PBMC	Asymptomatic	Central Italy	2000	80	F	C	Undetectable	
44 K	PBMC	Asymptomatic	Central Italy	2000	61	M	C	Undetectable	

### Laboratory procedures

#### Anti-HHV-8 antibodies

Anti-HHV-8 antibody titres have been assessed in sera by an immunofluorescence based assay (IFA) using BCBL-1 cells as substrate, as previously described [6]. Briefly, cells were plated on slides either untreated or after 72 hrs incubation with 12-O-tetradecanoyl-phorbol-13 acetate (20 ng/ml, TPA, Sigma, St Louis, MO) to induce the expression of antigens associated to the lytic cycle.

#### HHV-8 viral load

Extraction of DNA from biological samples was performed by a semi-automated procedure (NucliSens Easy MAG, Biomérieux, France). To establish viral load, DNA was isolates from the sera and HHV-8 genome detection and quantitation (genome equivalents gEq/mL), was performed by a commercial kit (HHV8 Q-PCR Alert, Nanogen Advanced Diagnostics, Italy), according to the manufacturer instructions. The procedure involved a real time amplification reaction for a gene region that codifies the protein of the HHV-8 capsid gene (ORF26).

#### ORFK1 sequencing

Viral DNA was extracted from peripheral blood mononuclear cells (PBMC) and from paraffin-embedded sections of tissue biopsies, performed for histological analysis, by the described semi-automated procedure (Nuclisens Easy MAG, Biomerieux SA, FR). The ORFK1 was amplified by nested PCR. The first amplification round was performed with specific primers [33]: forward outer primer, 5’- GTT CTG CCA GGC ATA GTC-3’; revers outer primer AAT AAG TAT CCG ACC TCA T. PCR products were subsequently amplified by forward inner primer 5’-GCG GTT TGC TTT CGA GG-3’and reverse inner primer 5’-AGA TAC CAC ACA TGG TT-3’ to obtain a 679 bp fragment including the two hypervariable regions VR1 (aa 54–93) and VR2 (aa 191–228) [33,39].

The cycling conditions of the nested PCR were set as follows. First run initial denaturation: 94°C for 120 sec; amplification: 35 cycles (94°C for 30 sec, 50°C for 60 sec, 72°C for 120 sec); extension: 72°C for 5 min. Second run initial denaturation: 94°C for 120 sec; amplification: 29 cycles (94°C for 30 sec, 50°C for 45 sec, 72°C for 120 sec); extension: 72°C for 5 min. The annealing temperature was set at 50°C for both for outer and inner primers.

Each PCR contained a blank (distilled water instead of DNA templates) and a positive control (HHV-8 DNA isolated from BCBL-1). The reaction products were resolved by electrophoresis on 1.5% agarose gel, stained with GelRed Nucleic Acid Gel Stain (Biotium, Inc. 3159, Corporate Place, Hayward, CA) and subsequently purified by PCR clean-up protocol to remove salts, enzymes, and other PCR residuates (NucleoSpin Extrct II, Macherey-Nagel, Germany). The purified DNA samples were sent to Eurofins MWG Operon, Ebersberg, Germay, for sequencing.

### Genetic analysis

#### Datasets

Two datasets were built. The first dataset contained 27 sequences of the ORFK1 gene, including VR1 and VR2 segments, plus 11 specific reference sequences. All reference sequences were downloaded from the National Centre for Biotechnology Information (http://www.ncbi.nlm.nih.gov/). Reference sequences (genotype A: JN800486.1, AF151688.1, JN800487.1, AF130284.1, KF781665.1, GU097427.1; genotype C: FJ866517.1, DQ394064.1, DQ394068.1, DQ394038.1, DQ394054.1) were selected on the basis of the following criteria: 1) sequences already published in peer-reviewed journals; 2) no uncertainty about the genotype assignment. The second dataset contained 27 sequences of HHV-8 K1 from the study population and it was used to estimate the mean evolutionary rate and to perform the time-scaled phylogeny.

#### Likelihood mapping

The phylogenetic signal of each sequence dataset was investigated by means of the likelihood mapping analysis of 10,000 random quartets generated using TreePuzzle [41].

Groups of four randomly chosen sequences (quartets) were evaluated. For each quartet, the three possible unrooted trees were reconstructed using the maximum likelihood approach under the selected substitution model. The likelihood of each topology was estimated with the maximum likelihood method and the three likelihoods are reported as a dot in an equilateral triangle (the likelihood map). Three main areas in the map could be distinguished: the three corners representing fully resolved tree topologies, i.e. the presence of treelike phylogenetic signal in the data; the center, which represents stalike phylogeny, and the three areas on the sides indicating nework-like phylogeny, i.e. presence of recombination or conflicting phylogenetic signals. Extensive simulation studies have shown that > 33% dots falling within the central area indicate substantial star-like signal, i.e. a star-like outburst of multiple phylogenetic lineages [41].

#### Phylogenetic analysis

The sequences of all datasets were aligned using Clustal X as already described [42] and manually edited by Bioedit [43]. Subtype of HHV8 sequences was determined uploading sequences individually into the Bioafrica Oxford HHV8 Automated Subtyping Tool [44] (http://bioafrica.mrc.ac.za/rega-genotype/html/indexhhv8.html) and confirmed by phylogenetic analysis. The maximum likelihood (ML) phylogenetic tree was generated with the HKY + I + G model of nucleotide substitution, by using Phyml v 3.0. The evolutionary model was chosen as the best-fitting nucleotide substitution model in accordance with the results of the hierarchical likelihood ratio test (HLRT) implemented in Modeltest software version 3.7 [45]. The statistical robustness and reliability of the branching order within the phylogenetic trees was confirmed by the bootstrap analysis and considering as significant statistical support a bootstrap value > 70%.

#### Bayesian phylogenetic analysis: evolutionary rate estimate and dated tree

Bayesian Phylogenetic analysis was conducted by means of MrBayes using HKY as a model of nucleotide substitution. A Markov Chain Monte Carlo (MCMC) search was made for 10 × 10^6^ generations using tree sampling every 100th generation and burn-in fraction of 25% [46,47].

The dated tree and the evolutionary rate of the second dataset were co-estimated by using a Bayesian MCMC approach (Beast v. 1.7.4) implementing a HKY + Invariant + Gamma model using both a strict and an uncorrelated log-normal relaxed clock model [48,49].

As coalescent priors, were compared four parametric demographic models of population growth (constant size, exponential, logistic growth, expansion) and a Bayesian skyline plot (BSP, a non-parametric piecewise-constant model). The best fitting models were selected by means of a Bayes factor (BF, using marginal likelihoods) implemented in Beast.

In accordance with Kass and Raftery [50] the strength of the evidence against H_0_ was evaluated as follows: 2lnBF < 2 = no evidence; 2–6 = weak evidence; 6–10 = strong evidence; and >10 = very strong evidence. A negative 2lnBF indicates evidence in favour of H_0_. Only values of ≥ 6 were considered significant.

The MCMC chains were run for at least 100 million generations, and sampled every 10,000 steps. Convergence was assessed on the basis of the effective sampling size (ESS). Only ESS values of > 250 were accepted. Uncertainty in the estimates was indicated by 95% highest posterior density (95% HPD) intervals. Statistical support for specific clades was obtained by calculating the posterior probability of each monophyletic clade.

### Selective pressure analysis

Comparison of relative fixation rates of synonymous (silent) and nonsynonymous (amino acid-altering) mutations provides a means for understanding the mechanisms of molecular sequence evolution. The nonsynonymous/synonymous rate ratio (ω = dN/dS) is an important indicator of selective pressure at the protein level, with ω = 1 meaning neutral mutations, ω < 1 purifying selection, and ω > 1 diversifying positive selection [51,52].

The CODEML program implemented in the PAML 3.14 software package (http://abacus.gene.ucl.ac.uk/software/paml.html) [51] was used to investigate the adaptive evolution of the K1 of HHV-8 Virus.

Six models of codon substitution: M0 (one-ratio), M1a (nearly neutral), M2a (positive selection), M3 (discrete), M7 (beta), and M8 (beta and omega) were used in this analysis [52]. Since these models are nested, we used codon-substitution models to fit the model to the data using the likelihood ratio test (LRT) [53]. The discrete model (M3), with three dN/dS (ω) classes, allows ω to vary among sites by defining a set number of discrete site categories, each with its own ω value. Through maximum-likelihood optimization, it is possible to estimate the ω and P values and the fraction of sites in the aligned data set that falls into a given category. Finally, the algorithm calculates the a posteriori probability of each codon belonging to a particular site category. Using the M3 model, sites with a posterior probability exceeding 90% and a ω value > 1.0 were designated as being “positive selection sites” [54]. The site rate variation was evaluated comparing M0 with M3, while positive selection was evaluated comparing M1 with M2. The Bayes empirical Bayes (BEB) approach implemented in M2a and M8 was used instead to determine the positively selected sites by calculating the posterior probabilities (P) of ω classes for each site [54]. It is worth noting that PAML LRTs have been reported to be conservative for short sequences (e.g. positive selection could be underestimated), although the Bayesian prediction of sites under positive selection is largely unaffected by sequence length [55,56]. The dN/dS rate (ω) was also estimated by the ML approach implemented in the program HyPhy [57]. In particular, the global (assuming a single selective pressure for all branches) and the local (allowing the selective pressure to change along every branch) models were compared by likelihood ratio test (LRT). Site-specific positive and negative selection were estimated by two different algorithms: the fixed-effects likelihood (FEL), which fits an ω rate to every site and uses the likelihood ratio to test if dN = dS; and random effect likelihood (REL), a variant of the Nielsen–Yang approach [55] which assumes that a discrete distribution of rates exists across sites and allows both dS and dN to vary independently site by- site. The three methods have been described in more detail elsewhere [58]. In order to select sites under selective pressure and keep our test conservative, a P value of ≤ 0.1 or a posterior probability of ≥ 0.9 as relaxed critical values was assumed.

For evolutionary analysis, the reference sequence Accession Number DQ394068 was used to trace the exact position of the amino acids found under selection.

### Statistical analysis

To test the differences between the two main clades and clusters in the phylogenetic tree respect to KS development, and the association of amino-acid sequences with clinical settings, the Fisher’s exact test for the categorical variables was used. *P* values <0.05 were considered statistically significant. Calculations of all statistical tests were performed by using SPSS V.21 Software.

## Results

### Virological parameters

All subjects had antibodies against either latent or lytic phase HHV-8 antigens with the following median titers and ranges. Asymptomatics: 1:80 (1:20–1:640); KS patients: 1:640 (160–5120); eMF and MF: 1:80 (1:20 – 1:160). Thirteen out of 15 KS patients and all the 4 MF patients had detectable HHV-8 DNA (Table 1). In Table 1 are also shown data of viral genotyping, as assessed by the sequence analysis of the ORFK1 region. As expected, only type A or C viruses were found in the study population.

### Likelihood mapping

**Figure 1:**
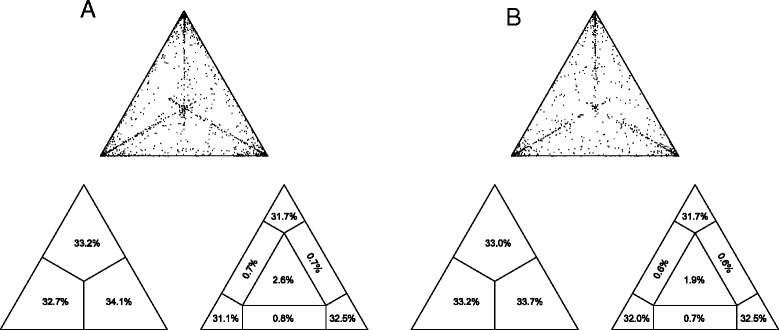
**Likelihood mapping analysis of K1 gene (VR1 and VR2 regions) sequences of HHV-8.** Each dot represents the likelihoods of the three possible unrooted trees for a set of four sequences (quartets) selected randomly from the data set: dots close to the corners or the sides represent, respectively, tree-like, or network-like phylogenetic signal in the data. The central area of the likelihood map, represents star-like signal (phylogenetic noise). Panel **A** and **B** represent the first and second data set, respectively.

The phylogenetic noise of each dataset was investigated by means of likelihood mapping (Figure 1A, B). The percentage of dots falling in the central area of the triangles was 2.6% for the first dataset, 1.9% for the second dataset; as none of the dataset showed more than 33% of noise, all of them were considered to contain sufficient phylogenetic signal.

### Phylogenetic analysis

**Figure 2:**
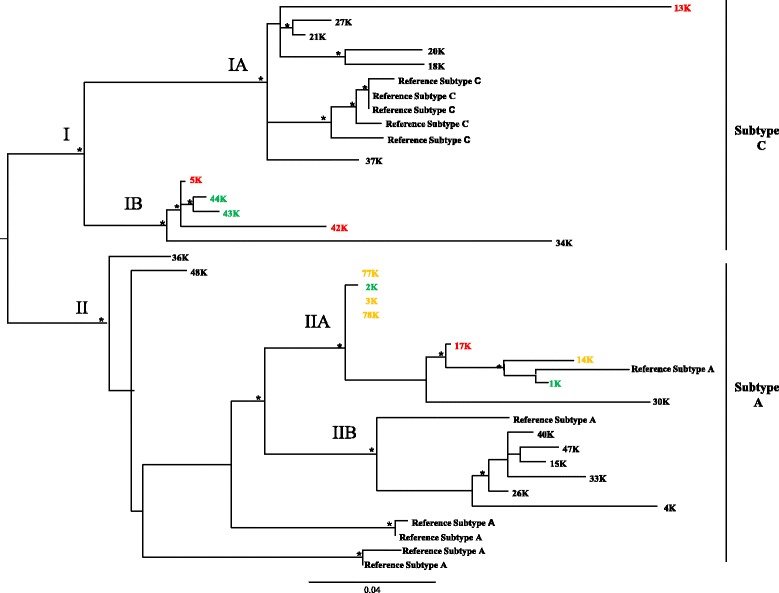
**ML phylogenetic tree for the first subtyping data set.** The tree was rooted by the midpoint rooting. Branch lengths were estimated with the best fitting nucleotide substitution model according to a hierarchical likelihood ratio test, and were drawn to scale with the bar at the bottom indicating 0.04 nucleotide substitutions per site. Sequences corresponding to HHV-8 isolates from different diseases are evidentiated in black (KS), red (MF), orange (eMF), green (no clinical symptoms). The asterisk (*) along the branches represents significant statistical significance for the clade subtending that branch (bootstrap value > 70%).

Maximum Likelihood phylogenetic tree of the first dataset showed 11 of 27 (41%) sequences classified as subtype C and 16 of 27 (59%) as subtype A (Figure 2) and distributed in two main clades (clade I and II respectively). Within the clade I two different statistically supported clusters were apparent (cluster IA and IB respectively). Cluster IA included 5 closely related isolates from KS patients and one isolate from MF patient, and cluster IB included isolates from 2 MF patients and 2 asymptomatic HHV-8 infected subjects and one isolate from KS patient. The same distribution occurred within the clade II where two main statistically supported clusters (cluster IIA and IIB respectively) were apparent. Cluster IIA, included isolates from 4 Early MF and 1 MF patients, 2 asymptomatic HHV-8 infected subjects and 1 KS patient; and cluster IIB included closely related isolates from KS patients.

The phylogenetic relationships among the different sequences of HHV-8 were supported by the bootstrap analysis with values >70%.

### Bayesian phylogenetic analysis: evolutionary rate estimate and dated tree

**Figure 3:**
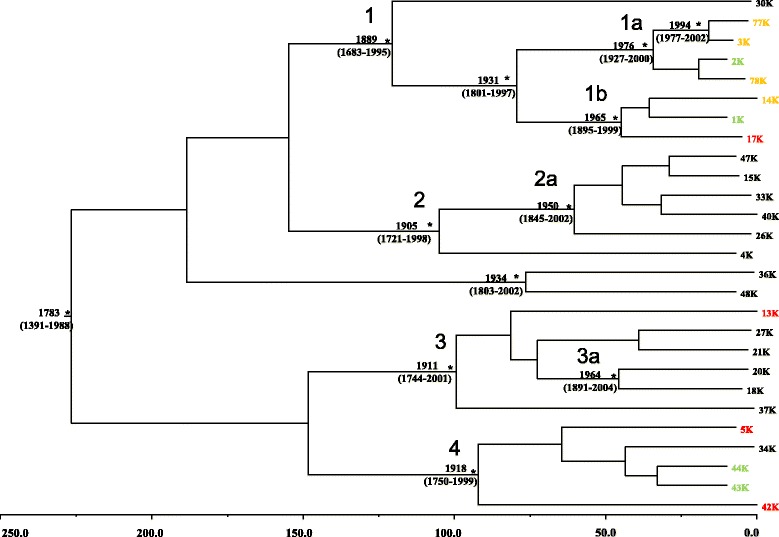
**Bayesian time-scaled tree of the 27 K1 HHV-8 gene sequences.** The numbers at the root and at the internal nodes represent the estimated date of the origin and the uncertainty indicated by 95% highest posterior density (95% HPD) intervals. Sequences corresponding to isolates from different diseases are evidentiated in black (KS), red (MF), orange (eMF), green (no clinical symptoms) The asterisks (*) along a branch represent significant statistical significance for the clade subtending that branch (posterior probability >97%). The line at the bottom represents time (in years).

The posterior probability >97% as statistical support for specific clades and cluster obtained in ML confirmed all the clades and clusters found (Data not shown). The Bayesian phylogenetic tree was built using the 27 isolates of HHV-8 (Figure 3). The estimated mean value of the HHV8 evolutionary rate was 6.42 × 10^−4^ substitution/site/year (95% HPD: 3.5706 × 10^−6^ - 1.4394 × 10^−3^).

The BF analysis showed that the relaxed clock fitted the data significantly better than the strict clock (2lnBF between the strict and relaxed clock was in favour of the second). Under the relaxed clock the BF analysis showed that the exponential growth model was better than the other models (2lnBF > 127). On the basis of this evolutionary rate, we estimated the time of the most common recent ancestor (tMRCAs) of the entire tree: in particular, the root of the tree had a tMRCA of 227 years before 2010 and dated back to the year 1783 (95% HPD: 1391–1988).

Four statistically supported clades (1, 2, 3 and 4) and a group of two sequences were evidenced. Clade 1 was dated back to the year 1889 (95% HPD: 1683–1995). One sequence labelled as 30 K was as out-group of the clade. Inside clade 1 two clusters (1a and 1b) were identified. Cluster 1a included one sequence labelled 2 K, isolated from an asymptomatic subject and three sequences isolated from eMF patients, which dated back to the year 1976 (95% HPD: 1927–2000). Cluster 1b dated back to the year 1965 (95% HPD: 1895–1999), included only three sequences, one isolated from an asymptomatic subject, labelled 2 K; one sequence isolated from a eMF patient, labelled 14KLP@10; and one sequence from a MF patient, labelled 17 K.

Clade 2, that was dated back to the year 1905 (95% HPD: 1721–1998) included only one statistically supported cluster (2a) where only closely related Kaposi sequences, isolated from tissue, appeared. These sequences dated back to the year 1950 (95% HPD: 1845–2002).

Clade 3, that was dated back to the year 1911 (95% HPD: 1744–2001) contained one statistically supported cluster (3a) including only two sequences isolated from KS and dated back to the year 1964 (95% HPD: 1891–2004).

Clade 4, that was dated back to the year 1918 (95%HPD: 1750–1999) contained one Kaposi sequence isolated from tissue labelled 34 K, sequences isolated from two asymptomatic subjects and one sequence isolated from MF patient, labelled 42 K.

The isolate group of two sequences that was dated back to the year 1934 (95% HPD: 1803–2002) contained only sequences isolated from KS patients.

### Evolutionary analysis

Selection pressure analysis on the K1 of HHV8 Virus revealed twelve statistically supported positively selected sites according to the reference sequence Accession Number DQ394068 (both by using HYPHY, PAML software) (Table 2). The α parameter of the γ distribution was < 1, shoving as this distribution has a characteristic L-shape, suggesting a nucleotide substitution rate heterogeneity across sites.

**Table 2:** **Selection analysis for the K1 gene of HHV-8 showing the effect of molecular sequence variation on amino-acid substitution**

Negatively selected sites*	102(Gln)		
(ω <1)**			
HYPHY software			
Positively selected sites*	44 (Glu,Ala,Thr,His,Ser,Pro)	58 (Ser,Phe,Trp,Leu,Val)	60 (Leu,Pro,Met,Ala)
(ω >1) **	62 (Gln, Asp, Lys,Glu,Thr,Arg)	66 (Leu,Phe,Thr,Asn,Ser,His)	66 (Leu,Phe,Thr,Asn,Ser,His)
HYPHY and PAML software	67 (Val,Pro,Ala,Gly,Ile)	68 (Gly,Val,Ala,Ser,Leu,Phe,Asp)	69 (Thr,Asp,Arg,Asn,Ile)
	70 (Ile,Phe,Leu)	71 (Ile,Ser,Val,Asn,Ala,Thr,His)	75 (Val,Thr,Ser,Leu)
	101 (Gly,Ala,Val,Arg)		

*Negatively selected sites and Positively selected sites are numbered according to amino acid position of K1 of HHV-8 Virus isolate accession number DQ394068.

**ω = dN/dS ratio of divergence at non-synonymous (nucleotide sites at which substitution causes an amino-acid change) and synonymous sites (nucleotide sites at which substitution does not causes an amino-acid change).

The average ω ratio ranges from 0.4214 to 3.099 among all models (Table 3), although in average the K1 of HHV8 virus, is under strong positive selection (ω > 1).

**Table 3:** **Likelihood Values and Parameter Estimates for the Selection Analysis of the K1 of HHV-8**

**Model**	**Free parameters**	**Log likelihood**	**Parameters estimates**	**Avg ω**
M0, one ratio	1	−2327.23	ω = 2.111	2.111
M1, neutral	1	−2336.44	p_0_ = 0.149; p_1_ = 0.851	1.000
M2, selection	3	−2186.37	p_0_ = 0.290; p_1_ = 0.553; p_2_ = 0.156; ω_2_ = 12.812	2.556
M3, discrete	5	−2184.71	p_0_ = 0.648; p_1_ = 0.218; p_2_ = 0.134; ω_0_ = 0.340; ω_1_ = 2.954; ω_2_ = 16.654	3.099
M7, beta	2	−2283.13	P = 0.005, q = 0.006	0.421
M8, beta, and ω	4	−2186.45	p_0_ = 0.843; P = 0.012; q = 0.005; p_1_ = 0.156; ω = 13.380	2.683

The discrete model (M3) fits the data significantly better than one ratio model (M0) with LRT statistic (2 Δ*l* = 285.02). The beta model (M7) is rejected when compared with the beta & ω model (M8) with LRT statistic (2 Δ*l* = 193.36). The discrete model (M3) indicates about 1% of sites are under positive selection with ω_2_ = 16.6545 (p < 0.05 d.f. = 4). Similarly in the beta & ω model (M8) there was 1.1% of sites are under positive selection with ω = 13.3798 (p < 0.05 d.f. = 2).

Only one negatively selected site, identified by FEL, for the K1 gene of HHV8 virus was showed in Table 2.

### Statistical analysis of disease association

The genotype A or C distribution, analyzed by contingency tables, was not significantly associated with geographic origin (Central vs South Italy) or with diagnosis of KS. The analysis of the association of clusters of ORFK1 with KS development by Fisher exact test showed a significant of cluster IIb (6 KS, 0 no KS), vs IIa (one KS, 7 no KS), p = 0.05; while the association of cluster Ia (5 KS, 1 no KS) vs Ib (1 KS, 4 no KS), did not reach a significant value, p = 0.08 (Table 4). The amino-acid substitutions at the 12 hypervariable codon sites for each sequence are described in Table 5. One similar sequence could be identified in 4/12 HHV-8 positive subjects without KS (3 K, 77 K, 78 K, 2 K) while it was not found in any KS sample (p = 0.03)

**Table 4:** **Contingency table analysis to assess the association between HHV-8 clusters and KS development**

**Clades**	**Clusters**	**KS**		**All**	**Fisher exact test**
		**No**	**Yes**		
I	IA	1 (16.7%)	5 (83.3%)	6 (100%)	P = 0.08
	IB	4 (100%)	1 (0.0%)	4 (100%)	
II	IIA	7 (87.5%)	1 (12.5%)	8 (100%)	P = 0.05
	IIB	0 (0%)	6 (100%)	6 (100%)

**Table 5:** **Amino–acid substitutions at the hypervariable codon sites of K1 protein of HHV-8**

**Sequence ID**	**Genotype**	**Diagnosis**	**AA codon 44**	**AA codon 58**	**AA codon 60**	**AA codon 62**	**AA codon 66**	**AA codon 67**	**AA codon 68**	**AA codon 69**	**AA codon 70**	**AA codon 71**	**AA codon 75**	**AA codon 101**
15 K	A	cKS	Ala	Phe	Pro	Asp	Phe	Pro	Val	Thr	Ile	Ser	Thr	Ala
18 K	C	cKS	Thr	Phe	Leu	Gln	Thr	Val	Val	Asp	Phe	Val	Ser	Val
20 K	C	cKS	Thr	Phe	Leu	Gln	Thr	Val	Ala	Arg	Leu	Val	Ser	Arg
21 K	C	cKS	Thr	Phe	Leu	Gln	Thr	Val	Val	Asn	Leu	Ile	Ser	Gly
26 K	A	cKS	Ala	Leu	Leu	Glu	Ser	Pro	Val	Thr	Ile	Ala	Thr	Ala
27 K	C	cKS	Thr	Phe	Leu	Glu	Thr	Val	Val	Asn	Leu	Ile	Ser	Gly
30 K	A	cKS	His	Trp	Leu	Lys	Asn	Ala	Ser	Thr	Ile	Asn	Leu	Ala
33 K	A	cKS	Ala	Phe	Leu	Glu	Phe	Pro	Leu	Thr	Ile	Ala	Thr	Ala
34 K	C	cKS	Glu	Ser	Leu	Gln	Leu	Val	Gly	Thr	Ile	Ile	Val	Gly
36 K	A	cKS	Ala	Leu	Leu	Lys	Thr	Ala	Val	Thr	Ile	Thr	Thr	Ala
37 K	C	cKS	Thr	Phe	Met	Gln	Thr	Val	Phe	Asn	Leu	Ile	Ser	Gly
40 K	A	cKS	Ala	Val	Leu	Glu	Phe	Pro	Val	Thr	Ile	Ala	Thr	Ala
47 K	A	cKS	Ala	Phe	Ala	Glu	Phe	Pro	Val	Thr	Ile	Ala	Thr	Ala
48 K	A	cKS	Ala	Trp	Leu	Arg	Thr	Val	Ala	Thr	Ile	Thr	Thr	Ala
4 K	A	cKS	Thr	Leu	Leu	Thr	His	Gly	Val	Asn	Leu	Thr	Thr	Ala
14 K	A	eMF	Thr	Trp	Leu	Lys	Thr	Ile	Asp	Ile	Ile	His	Leu	Ala
3 K	A	**eMF**	**Ser**	**Trp**	**Leu**	**Lys**	**Thr**	**Ile**	**Asp**	**Ile**	**Ile**	**Thr**	**Thr**	**Ala**
77 K	A	**eMF**	**Ser**	**Trp**	**Leu**	**Lys**	**Thr**	**Ile**	**Asp**	**Ile**	**Ile**	**Thr**	**Thr**	**Ala**
78 K	A	**eMF**	**Ser**	**Trp**	**Leu**	**Lys**	**Thr**	**Ile**	**Asp**	**Ile**	**Ile**	**Thr**	**Thr**	**Ala**
13 K	C	MF	Ser	Phe	Leu	Arg	Thr	Val	Val	Asn	Ile	Ser	Leu	Gly
17 K	A	MF	Pro	Trp	Leu	Lys	Thr	Ile	Asp	Ile	Ile	Asn	Leu	Ala
42 K	C	MF	Thr	Leu	Leu	Lys	Thr	Val	Val	Thr	Ile	Ile	Ser	Ala
5 K	C	MF	Thr	Leu	Leu	Lys	Thr	Val	Val	Thr	Leu	Ile	Ser	Gly
1 K	A	Asympt.	Thr	Trp	Leu	Lys	Thr	Ile	Asp	Ile	Ile	Asn	Leu	Ala
2 K	**A**	**Asympt.**	**Ser**	**Trp**	**Leu**	**Lys**	**Thr**	**Ile**	**Asp**	**Ile**	**Ile**	**Thr**	**Thr**	**Ala**
43 K	C	Asympt.	Thr	Leu	Leu	Met	Thr	Val	Asp	Thr	Leu	Ile	Ser	Gly
44 K	C	Asympt.	Thr	Leu	Leu	Lys	Thr	Ile	Val	Thr	Leu	Ile	Ser	Gly

Contingency tables and Chi square test suggest an association of amino-acid substitutions with HHV-8 infection and noKS: 4 sequences (boldface text)/12 vs 0/15 KS, p = 0.03 (Fisher).

## Discussion

KS is characterized by angioproliferative multifocal tumors of the skin or mucosa and less frequently the viscera, largely comprised of cells of endothelial origin with a unique spindled morphology, accompanied by a variable chronic inflammatory infiltrate [1]. Four different forms of KS are recognized: Classic (sporadic), African (endemic), acquired immune deficiency syndrome (AIDS)-associated (epidemic), and Transplant or immunosuppression-associated (iatrogenic) KS, all associated to prior infection with HHV-8 [1,2]. However, HHV-8 infection alone does not appear capable of inducing the development of KS. In fact, progression to KS is believed to depend on a complex and as yet incompletely understood interplay between HHV-8 and the host immune system that allows for the establishment of a tumor-promoting environment. Like all herpesviruses, HHV-8 establishes a life-long infection in the host that depends upon virus-encoded immune evasion genes and genes that influence cellular proliferation, survival, migration, angiogenesis and cytokine/chemokine production [2]. Different cells represent viral reservoirs in the infected host, including PBMC, endothelial cells, epithelial cells, and the KS spindle cells [1]. The host responds to persistent viral infection with a chronic inflammatory response, thereby facilitating events that, particularly in the context of immune suppression, might favor the viral oncogenic potentials. Understanding the dynamic relationship between host and viral factors that drives KS oncogenesis is central to the design of effective strategies to prevent tumor development. To better understand the HHV-8 contribution to KS development, we analyzed the sequences of the ORFK1 region of HHV-8 in tissue and PBMC samples from KS and non-KS HHV-8 positive subjects. These included either cKS patients or patients with a diagnosis of MF and eMF, according to the criteria by the International Society for Cutaneous Lymphomas [8], or clinically asymptomatic individuals. Although, the pathogenetic role of HHV-8 in MF and eMF remains to be established, we have previously found a significantly higher prevalence of HHV-8 infection in patients with the diagnosis of large plaque parapsoriasis (100%), a benign dermatosis with characteristics partially overlapping eMF, and in patients with MF (25%) as compared to that found in the general population (13,5%). In the same study we could amplify the ORF 26 coding for capsid viral protein even in those subjects with low anti-HHV-8 antibody titers, and detect ORF73 coded protein in tissue endothelial cell and some infiltrating lymphocytes, by immunohistochemistry. Nevertheless the role of virus in the development of the lesions is not ascertained [6].

The results of the present study show a statistically significant clustering of distinct viral isolates in two major clades corresponding to genotypes A and C. We did not observe any disease-specific segregation of A or C. Also the geographical area of origin was not associated to A or C viral subtypes. In fact, all subjects come from very close regions of central or south of Italy, with very similar rates of HHV-8 infection and KS incidence [3,59,60].

Within each of the major clades, however, HHV-8 isolates were clustered into two further distinguished subclades, one with significantly distinct distribution, according to the presence or the absence of KS, the other showing a distinct distribution, not reaching statistical significance (P = 0.08). This could suggest that KS patients are infected by HHV-8 strains differing in the ORFK1 region as compared to non-KS patients. This finding is further sustained by the bayesian evolutionary analysis showing a cluster (1a) of non-KS associated HHV8 strains.

These findings support the notion that viral genetic variations, as those associated to the ORF-K1 gene, may be associated to KS and might even play a part in KS development. In fact, K1 is a multifunctional protein that can constitutively activate multiple growth signaling pathways in infected cells [61]. The NH2-terminal region of K1 specifically interacts with the μ chains of BCR complexes, and this interaction retains BCR complexes in the endoplasmic reticulum, preventing their intracellular transport to the cell surface [62]. K1 was shown to functionally substitute for saimiri transforming protein (STP) of herpesvirus saimiri (HVS) and induce lymphoma in common marmosets [63]. Further, K1 induces B-cell lymphomas in transgenic mice through constitutive Lyn kinase activation, crucial for the production of VEGF and NF-kB activation [64,65], and induces MMP-9 and VEGF secretion from epithelial and endothelial cell lines [30,31]. The VEGF pathway appears implicated in both KS and MF pathogenesis [31,66].

## Conclusion

The main aim of this study was to analyze the variability rate of HHV-8, including samples from patients with cKS and from HHV-8 infected subjects without established HHV-8 associated diseases. The analysis of nucleotide variation of K1 and the consequent effect on amino-acid substitutions revealed 12 positively selected sites. One identical amino-acid sequence at the 12 codon sites under positive selection pressure could be identified in 4/12 HHV-8 positive subjects without KS, thus suggesting a role of HHV-8 in the development of the KS in the infected subjects. Although, the small number of cases and the long period of enrollment may have introduced significant bias on the conclusions of this study, our data encourage the planning of a larger study to to explore whether K1 polymorphisms may identify HHV-8 variants influencing the susceptibility to cKS in individuals with HHV-8 infection.
